# Genome Sequence of Lipopeptide- and Antioxidant-Producing Strain Bacillus velezensis NWUMFkBS10.5

**DOI:** 10.1128/MRA.00595-19

**Published:** 2019-07-11

**Authors:** Adetomiwa A. Adeniji, Olubukola O. Babalola

**Affiliations:** a Food Security and Safety Niche Area, Faculty of Natural and Agricultural Science, North-West University, Mmabatho, South Africa; University of Maryland School of Medicine

## Abstract

Candidate biocontrol agent Bacillus velezensis NWUMFkBS10.5 possesses unique genomic characteristics revealed by antiSMASH analysis and *in vitro* metabolomic elucidation. Besides its capability to produce antimicrobial lipopeptides, further *in silico* genome profiling predicted the presence of metabolic pathways for synthesizing antioxidants like lampranthin-2, miraxanthin V, and 2-decarboxybetanidin.

## ANNOUNCEMENT

The agroindustrial relevance of Bacillus velezensis species has become prominent in recent years (1–3), and more significantly, several strains producing beneficial lipopeptides with broad antimicrobial properties that could be exploited *in planta* and biotechnologically have been documented (4–7). With a view to providing an indigenous biocontrol agent against the etiological agents (e.g., Fusarium verticillioides, Fusarium culmorum, and Fusarium graminearum) of fusariosis, a worldwide disease that affects the quality and production of major cereal grains, we report here the draft genome sequence of an anti-*Fusarium* and antioxidant-producing B. velezensis strain isolated from the maize rhizosphere in the North West region of South Africa (8, 9). A pure culture of isolate NWUMFkBS10.5 was obtained from a distinct colony after 5 g of rhizospheric soil from the maize farm was cultured on HiCrome (Oxoid) selective *Bacillus* commercial agar plates following the manufacturer’s instructions. The isolate exhibited *in vitro* antifungal properties against F. graminearum and *F. culmorum* when grown on potato dextrose agar (Oxoid) (28°C for 7 days), and it produced active secondary metabolites (9).

The genome sequence conditions, protocols, assembly, annotation, data mining, and *in silico* analysis of this *B. velezensis* strain (NWUMFkBS10.5) have been previously documented by Adeniji et al. (9). Briefly, genomic DNA of the strain was extracted using the Zymo Research (ZR) soil microbe DNA miniprep genomic isolation kit. Sequencing of the DNA was done with the Illumina MiSeq reagent kit v2 microsystem at MR DNA (Shallowater, TX, USA), using in-house protocols. A Nextera DNA sample preparation kit (Illumina) was used in constructing the library, and thereafter, the Qubit double-stranded DNA (dsDNA) high-sensitivity (HS) assay kit (Life Technologies, Inc.) was used for the final library concentration. Determination of the average library size was done on an Agilent 2100 Bioanalyzer (Agilent Technologies). The library was then pooled and diluted to 12.0 pM, and the paired ends were finally sequenced using a 600-cycle v3 reagent kit (Illumina) with an average coverage of 50×. As previously reported by Adeniji et al. (9), the KBase platform (10) was employed for checking read quality (FastQC v1.0.1), read trimming (Trimmomatic v0.32), and adaptor sequence removal (Cutadapt v1.0.1). The assembly of reads into contigs was carried out on the KBase platform using Assemble with SPAdes v3.13.0. Contigs from KBase were then uploaded to the Rapid Annotations using Subsystems Technology v2.0 (RAST) and SEED Viewer v2.0 servers (11, 12), the PATRIC online server (v3.3.15) (13), and the NCBI Prokaryotic Genome Automatic Annotation Pipeline (PGAAP v4.2) (14) for automated annotation and comparison. Default settings were used for all of the bioinformatics analysis.

From the sequencing results with an average read length of 151 bp, a total of 15,010,234 paired-end reads and 7,505,117 clusters were obtained. The size of the NWUMFkBS10.5 genome and number of contigs were 3,964,473 bp and 37, respectively, with a G+C content of 46.39%. The *N*_50_ value of the raw sequences was 195,609 bp, and a total of 3,918 genes were annotated with PGAAP, including 3,701 coding genes, 51 rRNAs, 42 tRNAs, 5 noncoding RNAs (ncRNAs), and 166 pseudogenes. AntiSMASH 4.0.0rc1 (default parameters) (15) was used to predict the biosynthetic clusters (16 clusters) and bioproducts (bacillibactin, bacilysin, fengycin, difficidin, iturin, macrolactin, mersacidin, surfactin, and bacillaene) present in NWUMFkBS10.5. Based on phylogenomic characterization and Kyoto Encyclopedia of Genes and Genomes (KEGG) platform metabolic modeling (KEGG map, default settings), the strain was also reported to possess unique biosynthetic pathways capable of producing biomolecules (lampranthin-2, miraxanthin V, gomphrenin-1, 2-decarboxybetanidin, and celosianin-2) not previously documented for *B*. *velezensis* (8). This genome sequence should provide further avenues for the biotechnological manipulation of NWUMFkBS10.5 for possible applications outside agroindustry.

### Data availability.

This whole-genome shotgun project was deposited at DDBJ/ENA/GenBank under the accession number NZ_NITU01000037 and the assembly accession number GCF_002204665. The version described here is the first version, NZ_NITU01000037.1. The BioProject and BioSample designations for this project are PRJNA388288 and SAMN07174738, respectively.
